# Characterization of root-knot nematodes infecting mulberry in Southern China

**DOI:** 10.21307/jofnem-2020-004

**Published:** 2020-03-17

**Authors:** Pan Zhang, Hudie Shao, Chunping You, Yan Feng, Zhenwen Xie

**Affiliations:** 1 College of Agriculture and Biology, Zhongkai University of Agriculture and Engineering, Guangzhou, Guangdong, 510225, China; 2 College of Agriculture, Yangtze University, Jingzhou, Hubei, 434025, China

**Keywords:** Mulberry, Root-knot nematode, *Meloidogyne enterolobii*

## Abstract

China is one of the largest producers of mulberry in the world. With the development of the sericulture industry, several pests and diseases have occurred in rapid succession, chief among which is the root-knot nematode disease affecting mulberry. According to the China cocoon and silk exchange, cocoon prices have doubled since the beginning of 2009 and rose to 92,700 yuan ($135,770) per tonne in mid-April 2010. According to customs statistics, in the first eight months of 2011, China’s silk merchandise exports amounted to 2.39 billion yuan. In this study, sequencing of the rDNA-ITS and D2-D3 region of the 28S rRNA gene was combined with root-knot nematode morphological characteristics to identify the root-knot nematode infecting mulberry in the Guangdong, Guangxi, and Hunan provinces of China. This resulted in the identification of *Meloidogyne enterolobii* as the causal species of root-knot nematode infections in these regions. Importantly, the morphological data agreed completely with our molecular phenotyping efforts, indicating that rDNA sequencing could provide a more clear-cut and less labor-intensive means of characterizing root-knot nematode infections in the future. The differences between this study and the previous studies were discussed, as well as the damage degree, host species and influence scope of *M. enterolobii*.

Root-knot nematode disease has dramatically impacted *Morus alba* L. production in Japan, India, and Brazil (Hida and Zhu, 1985; Sujathamma et al., 2014; Paestakahashi et al., 2015). According to Wang and Chen (1989a, 1989b), root-knot nematodes cause mulberry leaf loss of 20 to 45%, with severe cases reaching over 75% in some fields (Wang and Chen, 1989a, 1989b). Leaf quality can also be negatively impacted by this pathogen. Root-knot nematodes on mulberry in Japan have previously been identified as *Meloidogyne mali*, *Meloidogyne hapla*, *Meloidogyne arenaria*, and *Meloidogyne incognita*, according to the morphological characteristics of female perineal pattern, male, and second instar larvae (Hida and Zhu, 1985). While in India (Schreiber et al., 2006; Kepenekcı et al., 2006; Sujathamma et al., 2014; Gnanaprakash et al., 2016) were identified as *Meloidogyne incongnita*. Paestakahashi et al. (2015) identified the root-knot nematodes of mulberry in Brazil as *Meloidogyne enterolobii* based on isozymes and the morphological characteristics of female perianal pattern, second instar larvae, and male spicule.

At present in China, the *Meloidogyne* that damages mulberry are mainly identified according to the morphological characteristics of perineal patterns, second-instar larvae, and male worms. It was reported by Xia (2004) that *Meloidogyne hapla* is mostly distributed in northern silkworm areas; *Meloidogyne arenaria* is mainly in Jiangsu, Zhejiang, and northern silkworm areas; *Meloidogyne incognita* is mostly spread in southern Zhejiang and Guangdong. Zhang et al. (1988) reported that *Meloidogyne incognita* was the nematode that caused the large-scale outbreak of *Meloidogyne* in the mulberry field of Sandland in Ankang City, Shanxi Province, and the four main species of *Meloidogyne* that damaged mulberry trees in Shanxi Province were *Meloidogyne arenaria*, *Meloidogyne incognita*, *Meloidogyne javanica*, and *Meloidogyne thamesi* (Wang and Chen, 1989a, 1989b). Tang et al. (2002) held that, the dominant populations of *Meloidogyne* on mulberry in Luliang County are mainly *Meloidogyne incognita* and *Meloidogyne arenaria*. Sun et al. (2004) reported that it was *Meloidogyne arenaria* that damaged mulberry in Qingzhou and other places in Shandong province. Qiao (2012) found that, the *Meloidogyne* parasitic on mulberry is *Meloidogyne incognita*. And Long et al. (2019) identified that, the *Meloidogyne* on mulberry in Hainan is *Meloidogyne enterolobii*.

Traditional identification methods are mainly based on morphology, especially the perineal pattern, which is sometimes inexact due to its large variability. Recent advances in molecular and morphological characterization have enabled better classification of root-knot nematodes. This strategy was employed to identify mulberry root-knot nematode in Hainan province as *Meloidogyne enterollobii* (Zhuo et al., 2008). In order to overcome the shortcomings of previous research that exclusively used morphology, we combined morphological characteristics with molecular biology to identify the causal pathogen of mulberry root-knot nematode disease in South China.

## Materials and methods

### Sampling

Samples were collected from rhizosphere soil and mulberry root-knots in six locations in South China, including Huadu City of Guangdong province, Guangzhou City of Guangdong province, Zhanjiang City of Guangdong province, Shaoguan City of Guangdong province, Nanning city of Guangxi province, and Changsha City of Hunan province. Samples were placed in sealed plastic bags, which were then placed in sample boxes and stored at 4°C before further analysis, in order to minimize changes in nematode populations.

### Nematode extraction

Male nematode was extracted from soil samples using the method described in Zeng (2012) study. Using a dissecting microscope, females and egg masses were isolated from infested roots with a scalpel and a nematological needle.

### Morphological observation

Individual nematodes were picked and heat-killed, then fixed in FG solution (containing 1 mL glycerol, 10 mL formalin, and 89 mL distilled water). The specimens were added slowly into glycerol and mounted on microscope slides. Measurements were made with a stage micrometer of Nikon microscope. Morphometric data were processed using Excel software. Images of key morphological features were taken using a Nikon DS-Fi1 attached to a Nikon ECLIPSE 80i microscope and processed using Photoshop CS5.

### Polymerase chain reaction and sequencing

A female was identified and separately placed in 5 μL of worm lysis buffer (WLB) containing proteinase K for DNA extraction (Williams et al., 1992). DNA samples were stored at −20°C.

To amplify the ITS region, we used primers 5367 (5′-TTGATTACGTCCCTGCCCTTT-3′) and 26s (5′-TTTCACTCGCCGTTACTAAGG-3′) described by Vrain et al. (1992). PCR reactions contained 12.5 μL 2× PCR buffer for KOD FX, 5 μL 2 mM dNTPs, 1 μL of each primer, 2 μL of isolated DNA, and distilled water up to 25 μL. The amplification was carried out in a lab cycler (Applied Biosystems) using the following program: initial denaturation at 94°C for 4 min; 35 cycles of denaturation at 94 °C for 1 min, annealing at 55 °C for 1 min, and elongation at 72 °C for 2 min; followed by a final extension at 72 °C for 10 min.

The D2-D3 region of the 28S gene was amplified with primer D2A (5′-ACAAGTACCGTGAGGAAAGTTG-3′) and D3B (5′-TCGGAAGGAACCAGCTACTA-3′) described by Sturhan et al. (2006). PCR reaction conditions were the same as those described for the ITS region.

### Sequence and phylogenetic analysis

The sequences obtained were submitted for a search in GenBank using the BLAST algorithm. Sequences for each gene were then aligned with corresponding published gene sequences using ClustalX 1.83 with default parameters. BI (Bayesian inference) analysis under the GTR + I + G model was initiated with a random starting tree and was run with four chains for 1.0 × 10^6^ generations. The Markov chains were sampled at intervals of 100 generations. Two runs were performed for each analysis. The log-likelihood values of the sample points stabilized after approximately 10^4^ generations. The topologies were used to generate a 50% majority rule consensus tree. Posterior probabilities (PPs) are given on appropriate clades. Sequence differences between samples were calculated with PAUP* 4b 10 (Cummings, 2004) as an absolute distance matrix and the percentage was adjusted for missing data.

## Results

### Morphology of root-knot nematodes from mulberry

**Figure 1:**
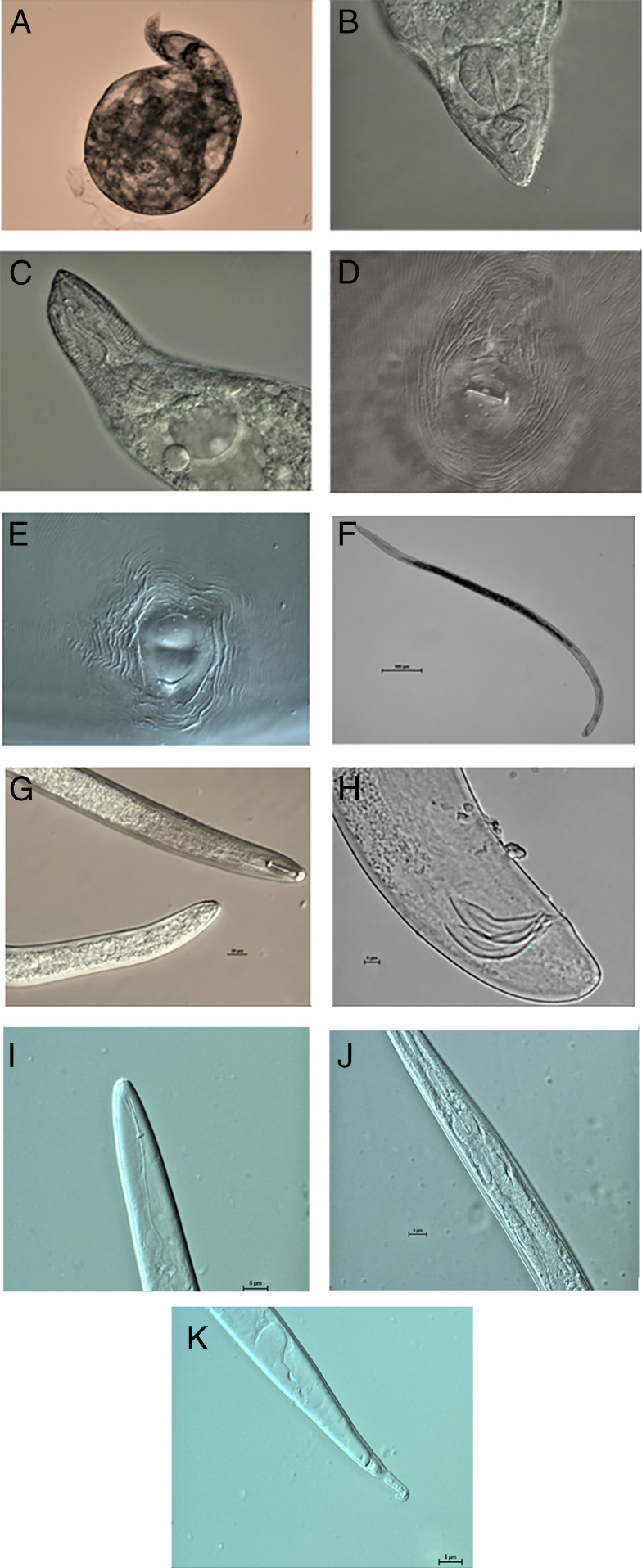
Representative morphological characteristics of *M. enterolobii.* (A, whole female; B, **C**, female head; D, E, female perineal pattern; F, male entire; G, male head and tail; H, male posture; I, head of G second instar larva; J, middle of second instar larva; K, tail of second instar larva).

The morphology of the root-knot nematode population isolated from mulberry trees is shown in Figure 1. The morphometric measurements are shown in Tables 1-3.

**Table 1.** Measurements of females of *Meloidognye* sp. in mulberry root knot.

Character	Range	Mean ± SD
Linear (μm)		
*n*	20	20
Body length	550 – 850	676 ± 97.9
Body width	403 – 750	491.4 ± 67.0
Neck length	3.6 – 4.3	3.9 ± 0.2
Stylet length	12 – 19	14.7 ± 1.7
Stylet knob height	1.8 – 2.9	2.3 ± 0.3
Stylet knob width	3.9 – 5.4	4.7 ± 0.5
DGO	2 – 4	2.9 ± 0.6
Excretory pore to Head end	41.3 – 79.6	55.8 ± 10.2
*a*	0.94 – 1.93	1.50 ± 0.3

**Table 2.** Measurements of males of *Meloidognye* sp. in mulberry root knot.

Character	Range	Mean
Linear (μm)		
*n*	20	20
Body length	1,500.0 – 1,910.6	1,661.5 ± 109.7
Body width	36.8 – 48.0	32.7 ± 15.4
Tail length	8.2 – 19.5	11.6 ± 2.3
Stylet length	21.1 – 24.7	16.0 ± 3.9
Stylet knob height	2.4 – 3.8	3.1 ± 0.4
Stylet knob width	4.1 – 5.6	4.6 ± 0.4
DGO	3 – 5	3.6 ± 0.6
Excretory pore to head end	158.9 – 206.1	174.4 ± 12.4
Spicule length	27.3 – 31.3	29.0 ± 1.2
Testis length	758.0 – 1,050.0	834.5 ± 64
*a*	34.0 – 44.7	37.5 ± 1.9
*c*	71.1 – 170.4	110.8 ± 31.1

**Table 3.** Measurements of 10 juveniles of *Meloidogyne enterolobii* n. sp.

Character	Range	Mean
Linear (μm)		
*n*	20	20
Body length	360 – 440	399.7 ± 21.3
Body width	13 – 17	13.6 ± 0.7
Tail length	40.5 – 62.4	50.8 ± 5.7
Excretory pore to head end	83.5 – 97.5	89.8 ± 3.5
Stylet length	10.4 – 12.8	10.9 ± 0.7
Stylet knob height	1.6 – 1.7	1.60 ± 0.00
Stylet knob width	2.4 – 3.0	2.60 ± 0.2
DGO	2 – 5	3.1 ± 0.7
*a*	24 – 29	26.7 ± 1.5
*c*	6.2 – 10.0	7.5 ± 0.9

### Female

The female body is white, pear-shaped to globular, with a prominent neck of variable size, and no posterior protuberance (Fig. 1A). The head region was not distinctly set off from the neck. Position of excretory pore is variable, and often found near the metacorpus (Fig. 1B). Cuticular body annulations become progressively finer posteriorly. Stylet is slender and its conical portion slightly curved dorsally, tapering toward tip. Contains cylindrical shaft, posterior end often enlarged (Fig. 1B). The conical portion is slightly curved dorsally, tapering toward the tip, with a cylindrical shaft that is posterior and often enlarged (Fig. 1C). Perineal pattern usually oval shaped, with coarse and smooth striae. The dorsal arch is moderately high to high, often rounded, with no lateral lines on perineal pattern for most females, or only one or two unclear lateral lines for a few samples (Fig. 1D,E).

### Male

The body is translucent white, vermiform, and tapered at both ends. It is small in size with a clear body ring (Fig. 1F). The head cap is highly rounded and slightly shrinking, with no ring pattern. Cephalic framework is moderately developed, with a distinct vestibule and extension (Fig. 1G). The stylet is robust with a straight cone. The boundary between the pole and the Base ball is clear (Fig. 1G). The Base ball of the stylet is large and the middle esophagus ball is fusiform. The tail is short and rounded, with a round base (Fig. 1G). The posture is slightly curved, while the base is round (Fig. 1H).

### J2

It has a body with translucent white and vermiform. The body ring is small and clear. The head slightly shrinks with fold marks. The labial disc is rounded and raised slightly above medial lips (Fig. 1I). The stylet is slender, with a straight cone, that is narrow and sharply pointed. The boundary with the rod is obvious. The distance of the DGO to the base of the stylet is long (3-5 μm). The mid-esophageal is bulbous shaped, with a clear valve (Fig. 1J). The Base ball of the stylet is clear, large, and oblong (Fig. 1I). There is a narrow tail, and the Hyaline tail terminus is clearly defined. The tail tip is obtuse, and has a 1 to 3 notches, with a dilated rectum (Fig. 1K).

The main morphological features are consistent with the description of the morphology of the ear worms of Yang and Eisenback (1983), Liao et al. (2001) and Long et al. (2015). This morphological similarity indicates that all six of the root-knot nematode populations are *Meloidogyne enterolobii*, but confirmation through molecular phenotyping is required to be certain.

### Molecular profiles and phylogenetic relationships

**Figure 2:**
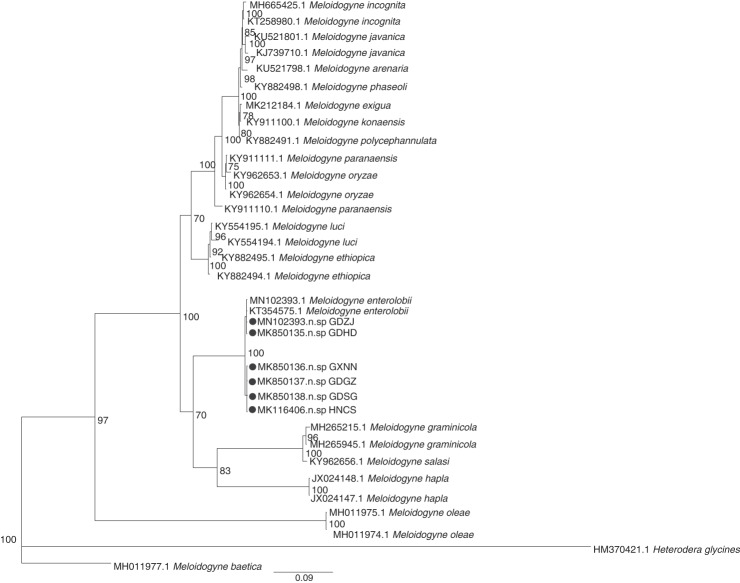
Phylogenetic relationships within root-knot nematodes on mulberry as inferred from Bayesian analysis of the *rDNA-ITS* gene sequences. Posterior probability values more than 70% are given on appropriate clades.

**Figure 3:**
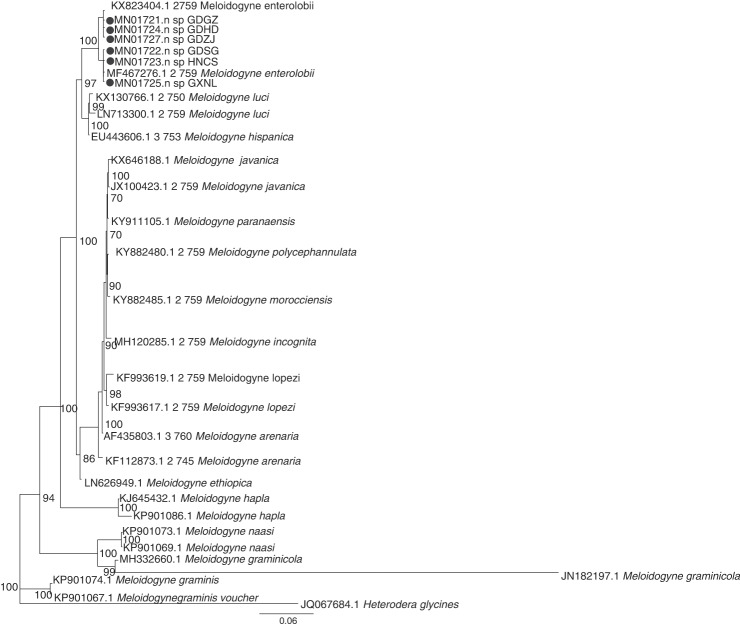
Phylogenetic relationships within root-knot nematodes on mulberry as inferred from Bayesian analysis of the D2-D3 region of the 28S gene sequences. Posterior probability values more than 70% are given on appropriate clades.

The primer pairs D2A/D3B and 26S/V5367 were used to amplify the D2-D3 region of the 28S and rDNA-ITS gene sequences of root-knot nematodes in the Guangdong, Guangxi, and Hunan province of China. The amplified products were 765 bp and 715 bp, respectively. The sequence of the amplified product was submitted to GenBank for a BLAST search, and the results revealed the highest similarity (99-100%) to sequences of *Meloidogyne enterolobii*. The phylogenetic tree inferred from rDNA-ITS (Fig. 2) suggests that MK850135, MN102393, and *Meloidogyne enterolobii* (KX823381.1 and KX823382.1) are in the monophyletic clade, while MK850136, MK850137, MK850138, and MK116406 are in another monophyletic clade in relation to a population of *Meloidogyne enterolobii* (KX823381.1 and KX823382.1). The phylogenetic tree inferred from the D2-D3 region of the 28S rRNA (Fig. 3) indicated that MN01721, MN01724, MN017127, and *Meloidogyne enterolobii* (MF467276.1) are in the monophyletic clade, while MN01722, MN01723, MN01725, and *Meloidogyne enterolobii* (KX823404.1) are in another monophyletic clade.

## Discussion

*M. enterolobii* (also known as *Meloidogyne mayaguensis*) was first discovered on *Euterolobium coutortisiliquum* in Hainan Province (Yang and Eisenback, 1983). It has become one of the most threatening pathogenic nematodes in both tropical and subtropical regions of the world, with estimated potential yield loss of 20% (Zhuo et al., 2008).

In this study, morphological characteristics and sequencing of the D2-D3 region of the 28S gene and rDNA-ITS region confirmed that the causal pathogen of mulberry root-knot nematode disease in Guangdong, Guangxi, and Hunan was *M. enterolobii*, which is consistent with the results reported by Paestakahashi et al. (2015) in Brazil and by Long et al. (2019) in Hainan province of China based on morphological and molecular characteristics. However, Wang and Chen (1989a, 1989b) identified pathogens of mulberry root-knot nematode disease in Shanxi as *Meloidogyne arenaria*, *Meloidogyne incognita*, *Meloidogyne javanica*, and *Meloidogyne thamesi*, while Tang et al. (2002) identified pathogen of mulberry root-knot nematode disease as *M. incognita* and *Meloidogyne arenaria* in Yunnan based on the female perineal pattern, anal characteristics, male posture, tail length, and stylet of J2. The reason for the inconsistency with the previous results may be that the variable perineal pattern of the female (Zhuo et al., 2008). It is easy to misidentify *M. enterolobii* as other species of *Meloidogyne* based only on the morphological characteristics. Castillo et al. (2001) identified pathogen of mulberry root-knot nematode disease as *Meloidogyne arenaria* by means of isozyme and morphological characteristics of female perineal patterns, J2, and male spicule. The reason for this difference may be related to different identification methods and different geographical positions.

*M. enterolobii* can infect many hosts, including: *Glycine max (Linn.) Merr*, *Zea mays* L., *Gossypium* spp., *Nicotiana alata Link et Otto*, *Psidium guajava Linn*, *Litchi chinensisSonn*., *Citrullus lanatus*, *Lycopersicon esculentum Mill*., *Ipomoea batatas (L.) Lam*., *Capsicum annuum L*., *Vigna sinensi (L.) Sav*., *Cajanuscajan (Linn.) Millsp*., *Cucurbita moschata*, *Aquilaria sinensis (Lour.) Spreng*., *Syzygium aromaticum*, *Ziziphus jujuba Mill*., *Piper nigrum L.*, *Cucumis melo var. saccharinus*, *Ipomoeaaquatica Forssk*, and *Morinda citrifolia* (Rammah and Hirschmann, 1988; Liao et al., 2001; Brito et al., 2007). In recent years, *M. enterolobii* has gradually spread to the north (Wu and Zhang, 2019), while *M. enterolobii* is already widely distributed around the world, and has been reported in Africa, America and Europe (Yang, 2013). Therefore, it will be particularly important to understand the genetic diversity of the root-knot nematode in mulberry and the variation among populations in different geographical locations in order to effectively combat it in the future. Our results indicate that molecular phenotyping is a robust method to conclusively determine the causal agent of root-knot nematode infections.
